# Boosting the Sustainable Transformation of *Cornus mas* L. Stones Using a Hybrid Strategy Involving Microwave-Assisted Extraction

**DOI:** 10.3390/molecules31030525

**Published:** 2026-02-02

**Authors:** Stanislava S. Boyadzhieva, Flora V. Tsvetanova, Jose A. P. Coelho, Plamena Staleva, Mariana Kamenova-Nacheva, Sabina Taneva, Roumiana P. Stateva

**Affiliations:** 1 Institute of Chemical Engineering, Bulgarian Academy of Sciences, “Akad. G. Bonchev” Str. bl. 103, 1113 Sofia, Bulgaria; maleic@abv.bg (S.S.B.); florablue@abv.bg (F.V.T.); 2 Instituto Superior de Engenharia de Lisboa, Instituto Politécnico de Lisboa, Rua Conselheiro Emídio Navarro 1, 1959-007 Lisboa, Portugal; 3 Centro de Química Estrutural, Institute of Molecular Sciences, IST-ID Associação do Instituto Superior Técnico para a Investigação e Desenvolvimento, Universidade de Lisboa, 1000-043 Lisboa, Portugal; 4 Institute of Organic Chemistry with Centre of Phytochemistry, Bulgarian Academy of Sciences, Acad. Georgi Bonchev Str., bl. 9, 1113 Sofia, Bulgaria; plamena.staleva@orgchm.bas.bg (P.S.); mariana.nacheva@orgchm.bas.bg (M.K.-N.); sabina.taneva@orgchm.bas.bg (S.T.); 5 Research and Development and Innovation Consortium, Sofia Tech Park JSC, 111 Tsarigradsko Shose Blvd., 1784 Sofia, Bulgaria; 6 Centre of Competence “Sustainable Utilization of Bio-Resources and Waste of Medicinal and Aromatic Plants for Innovative Bioactive Products” (BIORESOURCES BG), 1000 Sofia, Bulgaria

**Keywords:** *Cornus mas* stones, central composite design, microwave-assisted extraction, bioactive compounds, antioxidant activity, fatty acids, gallotannins and ellagitannins

## Abstract

A hybrid two-route strategy for converting *Cornus mas* L. stones into bioactive and other high-value compounds was developed and thoroughly evaluated. In Route 1, microwave-assisted extraction (MAE) is applied directly to the stones biomass following an experimental design created with Design Expert 11. Route 2 involves Soxhlet *n*-hexane extraction of the raw biomass, followed by MAE of the resulting defatted residue. The efficiency of the two routes was evaluated by comparing total polyphenol, flavonoid, and saponin content (TPC, TFC, TSC) and antioxidant activity (AA) of all obtained extracts, the fatty acid composition of MAE (route 1) and Soxhlet *n*-hexane extracts, and the metabolite composition of MAE extracts recovered in Route 1 and Route 2. The series of analyses performed involved GC–FID fatty acid profiling and composition determination using HPLC-HRMS/MS. These analyses showed that Soxhlet oil yield was 4.00 ± 0.18% with low AA, whereas subsequent MAE extracts had higher TPC, TFC, and TSC and 1.7-fold higher ABTS values than those of MAE Route 1. The increased AA is likely a result of the higher overall phenolic content, especially the presence of the potent antioxidant methyl gallate, which was not detected in MAE Route 1 extract, and not identified in C. cherry stones until now. Our results show that the CCD-optimized hybrid strategy effectively maximizes the recovery of bioactive compounds, demonstrates the superior potential of Route 2 for obtaining antioxidant-rich extracts, and widens the extent of applications of the underused C. cherry stone biomass.

## 1. Introduction

*Cornus mas*—a shrub or a small tree from the dogwood family *Cornaceae*—includes about sixty-five known species [1], distributed mainly in the east and south regions of Europe and West Asia [2]. Only a few species of this genus produce useful fruits, among which the most important is *Cornus mas* L., commonly known as Cornelian cherry [3], which is usually abbreviated to C. cherry. C. cherry is slow-growing, long-living, and very adaptive, with fleshy and edible red fruits [2]. It is native to the warm and mild zones of Eurasia, with a Pontic and Mediterranean distribution—from the Pyrenees, France, Italy, and the Balkan Peninsula to Asia Minor (Turkey, the Caucasus) [4].

Nowadays, C. cherry is cultivated in the Americas (the USA, Canada, Chile), Turkey, and Azerbaijan, while in some European countries—Italy, France, Poland, the Czech Republic, Slovakia, and Ukraine, it is farmed only on a small scale [5,6]. In Bulgaria, C. cherry, as a cultivated plant, is represented by five officially registered cultivars and a variety of large-fruited forms, which as individual trees are mainly grown in backyards and villas, fruit nurseries, mixed orchards, and others. In recent years, there has been a tendency to create C. cherry orchards, but still there is no official program adopted and implemented [7].

The phytochemical composition of C. cherry is quite abundant, which highlights the wide variety of benefits it can offer. The composition, however, to a great extent, depends on the cultivar of the plant and its genotype. The functional properties of C. cherry have been the object of a number of in vitro and toxicological studies. For example, in their review published in 2016, Biswanath Dinda at al. [8] summarized the phytochemical, pharmacological, toxicological, and clinical studies of the fruits, leaves, barks, and flowers of C. cherry plant as well as their ethnomedicinal uses. About 101 compounds were identified, among which anthocyanins, flavonoids, and iridoids are the predominant representative groups. Novruzov et al. [9] analyzed the composition and antioxidant activity of ethanolic extracts of the fruits, leaves, and stones of *Cornus mas* L., grown in Azerbaijan, and showed that the highest content of anthocyanins was found in fruits and flavonoids and typical phenols in leaves, while stones were the richest in catechins.

The recent review of Cevic et al. [10] reported that the phytonutrients found in *C. mas* flowers, fruits, and leaves include flavonoids, procyanidins, anthocyanins, phenolic acids, monoterpenes, iridoids, triterpenes, carotenoids, vitamins, fatty acids, organic acids, carbohydrates, and minerals. For example, in C. cherry fruits, the major compounds are ascorbic acid and anthocyanins. The content of the latter in fruits is much higher than that reported for Brazilian blackberry, red raspberry, strawberry, blueberry, and sweet cherry, with the main anthocyanins being cyanidin and pelargonidin 3-O-glycosides [11]. On the other hand, leaves contain higher amounts of phenolic compounds than fruits, but no anthocyanins, while flowers are rich in phenolic compounds, flavonoids, and terpenes.

Most recently, Lidiková et al. [12] studied the mineral and bioactive content of the fruits of several cultivars of C. cherry. Neochlorogenic, chlorogenic, and caffeic acids, rutin, and vitamin C were identified in the 80% methanolic fruit extracts. The total polyphenol and anthocyanin content as well as antioxidant activity were determined. It was demonstrated that genetic influences on the bioactive and mineral composition are substantial, even though the different cultivars examined were grown under identical conditions.

The health benefits of the *Cornus* plant are plentiful [13]. Its leaves, ripe fruits, and barks have been used for medicinal purposes for centuries [14]—the fruits to treat dysentery and diarrhea, while the leaves are effective in the treatment of ringworm [8]. Tinctures made from either the leaves or the barks of the plant can be used as remedies for eczema, skin infections, intestinal parasites, veal skin, gout, etc. [15]. In traditional medicine, C. cherry is also used in the cases of excessive menstrual bleeding, excessive sweating, spermatorrhea, and to treat chronically sore backs and knees [16,17]. Bearing in mind that all parts of the plant contain tannins and consequently have astringent properties, they can also be used as a substitute for quinine [8].

In the review by Cevik et al. [10], the ethnomedical uses of *C. mas* were summarized and reported for 16 countries. It was shown that the main applications of the fruits, leaves, and stones, either directly or as extracts, fermented products, etc., include the treatment of cold, fever, heatstroke, cough, bronchitis, diabetes, urinary tract infections and inflammation, diarrhea, abdominal pain, ulcer, kidney stones and infections, cancer, sunstroke, loss of appetite, wounds, etc.

For all the above reasons, C. cherry was targeted in the EU program BaSeFood [18], in which the uses, composition, and traditional foods of local crops were investigated and compared in a cross-country perspective.

The number of *in vivo* and *in vitro* bioactivity studies on extracts recovered from C. cherry published in the literature is considerable [10]. Still, it is important to underline that the extracts were obtained predominantly from the fruits and some from the leaves and peels of C. cherry applying either ethanol, methanol, acetone, water, or mixtures, e.g., methanol + ethanol, methanol + acetone + formic acid + water, etc. However, just one study reported the antimicrobial activity of C. cherry stones’ methanol and ethanol extracts.

As is well known, not only the solvent but also the extraction technique and its operational parameters, particularly temperature and time, can significantly influence the recovery of various compounds, particularly thermolabile bioactives and secondary metabolites. Literature analysis shows that, to date, mostly conventional methods (e.g., Soxhlet) have been used, while the application of innovative techniques to transform various parts of C. cherry plant biomass into enriched extracts remains an open issue. For example, ultrasound-assisted, supercritical fluid, and subcritical water extractions were used to recover bioactive compounds predominantly from C. cherry fruits, see [19], while a microwave-assisted digestion technique to determine the major and minor elements in the whole fruits, mesocarps, and stones was used in [20]. In a recent publication, Enache et al. [21] discuss the state of the art of C. cherry fruit valorization and point out that a range of solvents have been used to extract bioactives from its biomass such as water, acetone, methanol, ethanol, and acidified water. Therefore, studies that examine the application of advanced techniques will help substantiate novel solutions for the sustainable obtainment of C. cherry extracts that contain bioactive substances without/with the lowest possible changes in their functional properties and with improved beneficial activity. Thus, their direct use in pharmaceutical, nutraceutical, and cosmetic applications, the production of functional foods, etc. will be facilitated and expanded.

Among the different parts of C. cherry tree, the stones, which are treated as waste by-products of fruit processing, are generally overlooked and ignored in investigations and studies. However, their weight, depending on the cultivar of the plant and its genotype, is in the range of 13.02–21.3% [22] or 16% on average of the total weight of the fruit. Furthermore, stones, as discussed by Przybylska et al. [23] and Spychaj et al. [24], are the least known morphological part of the plant, with limited knowledge about their composition and biological properties. Thus, Przybylska et al. [23] were the first to identify 37 compounds present in the Soxhlet ethanol extracts of the stones, among which were various gallotannins and monomeric, dimeric, and trimeric ellagitannins, and they reported their antioxidant activity and total phenolic content assays. In a recent contribution, the authors commented that the monomeric, dimeric, and trimeric derivatives of the bioactives gallic and ellagic acids are plentiful in both the fruits and stones of C. cherry [25].

Vidrih et al. [26] examined and reported the soluble solid, mineral, and fatty acid compositions of 10 genotypes of C. cherry stones and demonstrated that the nutritional value of their oil is similar to that of sunflower, corn, pumpkin, and cotton oils. Kazimierski et al. [27] who studied the nutritional and pro-health properties of C. cherry stones reported that the fatty acids extracted from the stones exhibit antimicrobial activity against Gram-positive and Gram-negative bacteria. They also referred to an earlier work of Krzyściak et al. [28] who showed that alcoholic extracts from the seeds exhibit high antibacterial activity against *S. aureus* and *E. coli*, and that their antifungal activity against *Candida albicans* is higher than that from the barks and fruits of the plant.

The current status of C. cherry stone transformation into high-value compounds encompasses the water infusions of roasted stones with distilled water at approximately 80 °C [29], Soxhlet ethanol extraction [23], supercritical fluid extraction at various pressures in the interval of 119–331 bar and various temperatures (36–64 °C) [30,31], and extraction with methanol and ethanol (40, 70, 80, 95%) [9].

In light of these considerations, the present study aims to develop and rigorously evaluate a hybrid two-route strategy for converting C. cherry stones into bioactive and other high-value compounds. In the first route, microwave-assisted extraction (MAE) is applied directly to the stone biomass according to an experimental design generated using Design Expert 11 (DE 11). The second route involves Soxhlet *n*-hexane extraction of the raw biomass, followed by MAE of the resulting defatted residue.

The efficiency of the two routes is evaluated by comparing the following:

(i) The total polyphenol, flavonoid, and saponin content (TPC, TFC, TSC) and antioxidant activity (AA) of all extracts obtained in Routes 1 and 2;
(ii) The fatty acid composition of MAE (route 1) and Soxhlet *n*-hexane (route 2) extracts;
(iii) The metabolite composition of MAE extracts recovered in Route 1 and Route 2.

As far as we are aware, this investigation is the first to apply a hybrid strategy integrating an advanced technique as MAE for the valorization of C. cherry stones biomass. Based on the results obtained and the analysis of the efficiency of Routes 1 and 2, new solutions to the sustainable valorization of C. cherry stones to bioactives and other high-value compounds could be substantiated. Thus, the perspectives on this available, abundant, but to a certain extent neglected and underutilized, biomass and its applications as nutraceuticals, ingredients in new food formulations, therapeutic agents, biofuel additives, etc. will be widened and refueled.

## 2. Results and Discussion

The results are presented in the next subsections, where the values are shown with their (±) standard deviations.

### 2.1. Extraction of C. cherry Stones—Route 1 of the Hybrid Strategy

In Route 1 of the hybrid strategy, MAE was used to recover extracts from the C. cherry stones. The experiments were performed following the scheme obtained by the Design Expert 11 (DE-11) program. It is an essential tool that can be successfully used in experiments involving multiple factors and responses. A central composite design (CCD) was used to examine the effects of those variables on the responses and to optimize the values with a minimum number of experiments. To apply CCD, time and power were set to 4.5 min and 90 W, respectively. This choice was based on the experience gained by the authors when the MAE of spent coffee grounds was examined and reported in a recent publication [32] and on the results of our preliminary test, which demonstrated the following: (*i*) extraction times longer than 5 min did not improve recovery; (*ii*) lower power levels (50–70 W) did not adequately penetrate the rigid structure (even powder) of the seed biomass, while higher power levels (110–150 W) resulted in localized overheating and the early degradation of analytes. The ratio of solvent to solute (solv/sol) in mL/g is denoted as A, and the ethanol–water ratio (Eth/water) in percentage (*V*/*V*) is denoted as B. The maximum and minimum values of the two factors (independent variables) and their respective coded values, as generated by the program DE-11, are shown in Table 1. Our proposed values are based on the coded values. Still, the CCD gave slightly different minimum and maximum values (Table 1), where the maximum value of 104.50% is actually of pure ethanol (100%).

As response functions, the yield (%), TPC, represented in mmol of gallic acid equivalents (GAE), TFC in mmol of catechin equivalents, AA of the DPPH and ABTS assays in mmol of Trolox equivalents (TE), and TSC as aescin equivalents (AesE), all by 100 g of extract, were chosen to characterize the obtained extracts. The results of the CCD tests are displayed in Table 2.

#### Statistical Analysis of the CCD Experimental Data

The experimental design identified the impact of the key factors A and B on the yield, TPC, DPPH, ABTS, TFC, and TSC of the extracts recovered. Statistical analysis was performed with analysis of variance (ANOVA) for each response. The statistical parameters for the independent variables, which are not average values, are detailed in Table A1 of Appendix A.

The analyses revealed that the models that best fit each response are distinct. Moreover, all the F-statistic values suggested that each model is significant, and the *p*-values are less than the alpha level of 0.05 (95%), meaning the results are statistically significant.

Extraction yield (%) was one of the primary response factors to the experimental design. The best model for this response was linear, with an F-value of 4.73, indicating significance. A non-significant lack of fit, with an F-value of 0.97, is considered good, indicating that it is not significant relative to the pure error.

The final equation, in terms of actual factors obtained, can be used to predict the response for the specified levels of each factor, where the levels should be expressed in the original units for each factor and presented as shown in Equation (1).

(1) $$Yield=14.73397+0.209735\left(Ratio\frac{Solv}{sol}\right)-0.015343\left(Ratio\frac{Et}{wat}\right)$$

**Figure 1:**
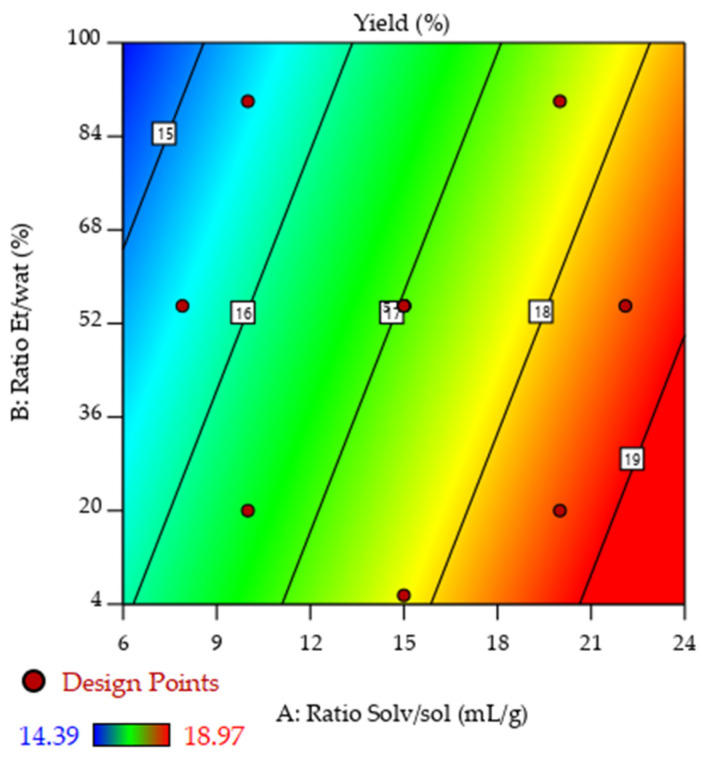
Response surface plot showing the effects of the solvent/solute and ethanol/water ratios on the C. cherry stones’ extraction yield.

Figure 1 represents the linear effect of the ratio solv/sol and Et/wat on the yield (%). It was demonstrated that, within the entire range of the ratio solv/sol, the increase in the solvent percentage in the mixture increased the yield. This was in complete agreement with the ANOVA analysis, where the parameter ratio Eth/water had a *p*-value < 0.1918 in contrast with the *p*-value of the ratio solv/sol, which was significant since it presents a value of 0.0209.

With regard to the other responses displayed in Table 2, an average value of 46.73 (mmol GAE/100 g) with a 95% confidence interval (CI) [44.57, 48.89] for TPC; 215.25 (mmol TE/100 g) with a 95% CI [207.27, 223.24] for DPPH, and 199.92 (mmol TE/100 g) with a 95% CI [190.56, 209.27] for ABTS were the best descriptions obtained from the ANOVA. Regarding TFC, a quadratic model is identified with an F-value of 4.07, indicating that the model is significant. The lack of fit has an F-value of 0.15, suggesting it is not significant relative to the pure error.

The equation, based on actual factors, predicts the response at specific levels of each factor, which should be expressed in their original units as shown in Equation (2).

(2) $$TFC=47.9461-2.1484\left(Ratio\frac{Solv}{sol}\right)+0.82828\left(Ratio\frac{Et}{wat}\right)\;\;-0.04107\left(Ratio\frac{Solv}{sol}\right)\times \left(Ratio\frac{Et}{wat}\right)+0.13024{\left(Ratio\frac{Solv}{sol}\right)}^{2}\;-0.00175{\left(Ratio\frac{Et}{wat}\right)}^{2}\;$$

For TSC, a quadratic model is also identified with an F-value of 5.60, indicating that the model is significant. The lack of fit has an F-value of 1.64, suggesting it is not significant relative to the pure error.

The equation is formulated using real factors to forecast responses at designated levels for each factor. It is important to express these factors in their original units, as demonstrated in Equation (3).

(3) $$TSC=-1.6169+1.3683\left(Ratio\frac{Solv}{sol}\right)+0.13436\left(Ratio\frac{Et}{wat}\right)\;\;-0.004477\left(Ratio\frac{Solv}{sol}\right)\times \left(Ratio\frac{Et}{wat}\right)-0.03612{\left(Ratio\frac{Solv}{sol}\right)}^{2}\;-0.001060{\left(Ratio\frac{Et}{wat}\right)}^{2}\;$$

**Figure 2:**
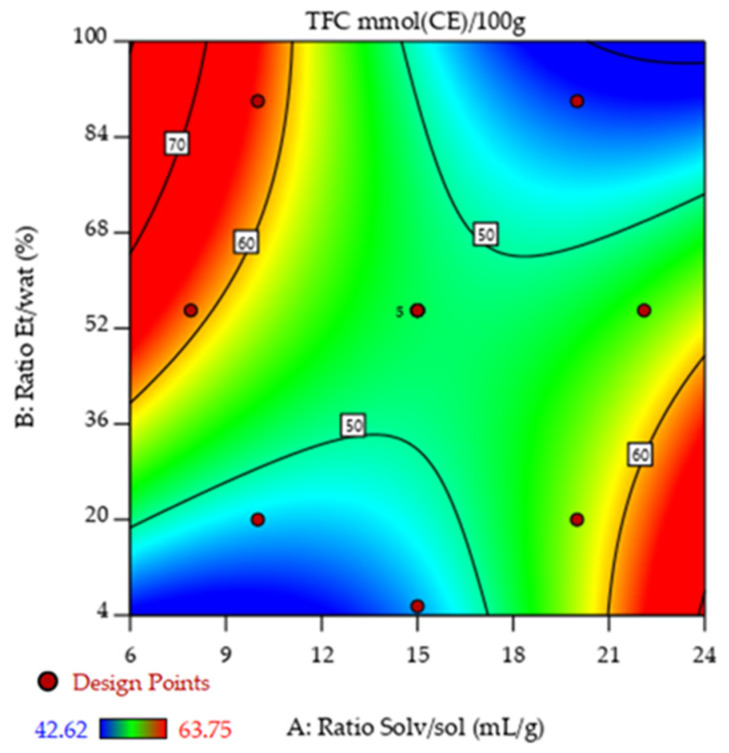
Response surface plot showing the effects of the solvent/solute and ethanol/water ratios on the TFC of C. cherry stone extracts.

**Figure 3:**
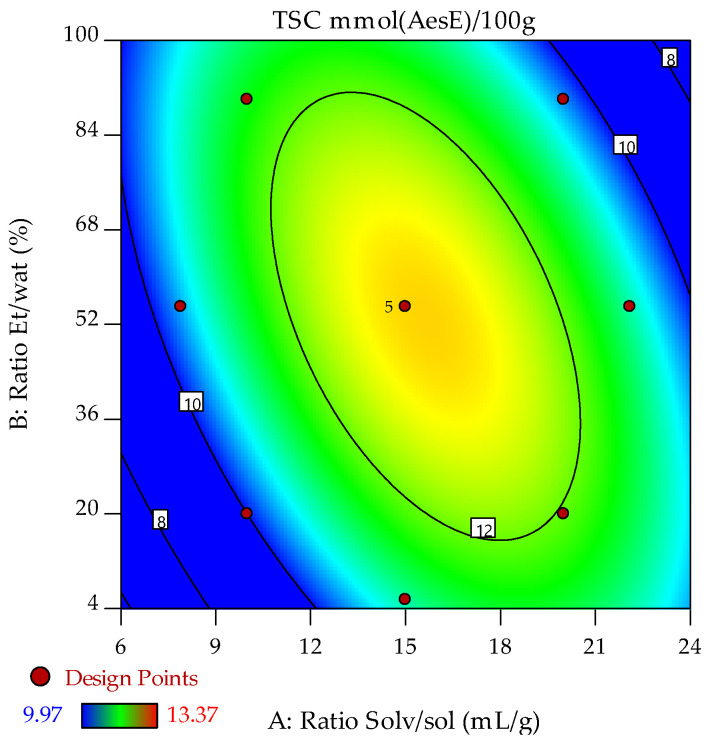
Response surface plot showing the effects of the solvent/solute and ethanol/water ratios on the TSC of C. cherry stone extracts.

Figure 2 and Figure 3 represent the results obtained from Equations (2) and (3) for TFC and TSC, respectively. In particular, Figure 2 illustrates the contour plot showing the combined effects of the solvent-to-solid ratio and ethanol/water ratio on TFC. The response surface reveals that both parameters significantly enhance flavonoid recovery, since a higher level of each consistently increases TFC. The highest extraction occurs when both factors are maximized simultaneously (red regions), emphasizing the need to optimize solvent volume and polarity together.

The contour plot in Figure 3 shows how TSC varies across the experimental design space. The highest TSC values are in the yellow regions, indicating optimal conditions for saponin extraction, while blue zones reflect lower efficiency. The distribution of design points confirms the reliability of the modeled response surface, linking observed gradients and optimal regions to experimental data.

It should be noted that TFC and TSC exhibit different extraction behaviors. TFC is sensitive to the solvent-to-solid ratio and ethanol concentration, showing sharp increases under high-solvent and high-ethanol conditions due to the semi-polar nature of flavonoids. In contrast, TSC has a smoother response, indicating greater tolerance to extraction conditions and less reliance on solvent polarity. Thus, TFC needs precise optimization, while TSC can be effectively extracted across a wider range of conditions, underscoring different priorities for maximizing each phytochemical.

Dupak et al. [33] extracted C. cherry stones with 80% ethanol for 2 h at a ratio of solute to solvent (mg/mL) of 100, and they reported DPPH assay and total polyphenols—7.84 mg TE/g and 29.61 mg GAE/g—which correspond to 3.13 mmol TE/100 g and 17.41 mmol GAE/100 g, respectively. Those values are significantly lower than ours (Table 2), as well as that for TFC—1.56 mg QE/g—which is on average also lower.

According to Spychaj et al. [29], the fresh stones exhibited high antioxidant potential, ranging from 166.48 to 509.74 μmol TE/g dw (16.65–50.97 mmol/100 g dw), which varied significantly depending on the cultivars. Conversely, roasted C. cherry stones retained between 43.6% (DPPH) and 97.2% (FRAP) of the antioxidant activity found in non-roasted stones, depending on the cultivar. The extracts were prepared as water infusions using distilled water at about 80 °C and from stones after roasting.

Przybylska et al. [23] determined the AA and TPC of extracts obtained from the milled stones of C. cherry, which were subjected to Soxhlet extraction using absolute ethanol for 3 h. The TPC had a value of 11466.53 mg GAE/100 g (67.40 mmol GAE/100 g), while the AA measured by ABTS and DPPH was 255.99 and 210.62 mmol TE/100 g, respectively. These values agree with our results but are higher than those reported in [33]. This discrepancy may be due to differences in the solvent used, the solvent-to-solute ratio, the method, or the extraction time, resulting in different results.

In a recent publication [24], the extraction of unroasted stones with 50% methanol was presented. The value reported for TPC was 3438.7 mg GAE/100 g and ABTS was 360.3 µmol TE/g. These results, recalculated for comparison purposes, are TPC of 20.21 mmol GAE/100 g and ABTS of 36.03 mmol TE/100 g, which are on average over 2 times lower than the TPC values achieved by us, and almost 6 times lower than the AA shown in Table 2.

### 2.2. Extraction of C. cherry Stones—Route 2 of the Hybrid Strategy

Route 2 involves the application of Soxhlet and MAE subsequently as explained briefly below:

Firstly, since C. cherry stones are known to be a valuable source of fatty acids [28], Soxhlet *n*-hexane was applied to recover the oil from the seed biomass (stage 1). The yield over a 4 h period was 4.00 ± 0.18%. The sample was tested using the DPPH assay, and a value of 28.91 ± 0.69 (mmol TE/100 g) was obtained, which is about seven times lower than the average DPPH value of the MAE extracts recovered in Route 1 (Table 2), which indicates the low AA of the Soxhlet extract. In principle, these extracts have lower total TPC, TFC, TSC, DPPH, and ABTS values because the solvent is non-polar and primarily dissolves lipophilic compounds such as fats and terpenes, which possess limited antioxidant activity. Hence, no further qualitative analyses (e.g., TPC) on that extract were performed.

On the other hand, ethanol and water as polar solvents efficiently extract polar and semi-polar bioactive compounds, such as phenolics, flavonoids, and saponins, which are potent antioxidants. Consequently, MAE with (ethanol + water) mixture results in higher TPC, TFC, TSC, DPPH, and ABTS values, as these compounds are more soluble in polar solvents and contribute more effectively to free radical scavenging as measured by the DPPH and ABTS assays.

The fatty acid composition of the oil was analyzed by applying GC-FID.

The resulting residue from the Soxhlet *n*-hexane extraction was dried, and on stage 2 it was subjected to MAE with a solvent-to-solid ratio of 15 and an ethanol-to-water ratio set at 55, which represents the central point of the experiments performed in Route 1 (Table 2). The solvent values at the interval end points, specifically between 5 and 100% ethanol, were also considered, as shown in Table 3.

Table 3 reveals that the yield achieved at the central point is commensurable but still lower than those reported in Table 2. The decline in yield is particularly pronounced at the end points. Still, these results are not unexpected, taking into consideration that the MAE was performed on the depleted biomass after the Soxhlet *n*-hexane extraction.

The qualitative analyses of the MAE extracts recovered showed that the TPC, TFC, TSC, and DPPH values are higher than those reported in Table 2. The difference is particularly pronounced when the ABTS values of Table 3 are compared to those displayed in Table 2. The former is approximately 1.7 times higher than the latter. These results underscore the efficiency of the hybrid strategy to impact the quality of the extracts obtained, particularly regarding their AA, which implicitly points to the better performance of Route 2 regarding the recovery of extracts with higher bioactive potential.

### 2.3. Analyses of Route 1 and 2 Extracts

As stated previously, in our study, we focus also on the rigorous evaluation of the effectiveness of the hybrid two-route strategy advocated, which is achieved by a comparison of the results of a series of compositional analyses of the extracts obtained, such as the following:

GC–FID fatty acid profiling performed on the MAE central point extract recovered in Route 1 and on the Soxhlet n-hexane extract of Route 2.

HPLC–HRMS/MS profiling carried out on the MAE extracts obtained in Route 1 and Route 2.

#### 2.3.1. GC-FID Ananysis

As mentioned above, one of the aims of our work is to compare the efficiency of Routes 1 and 2 with regard to recovering fatty acids from the seeds of C. cherry.

The reason behind integrating Soxhlet in the first stage of Route 2 is that, as known, n-hexane as a non-polar solvent is considered the best choice to recover neutral saponifiable lipids, while polar solvents like ethanol and water are preferable for polar lipids. On the other hand, the MAE extract of Route 1 is obtained by applying a mixture of polar solvents—55% ethanol + 45% water. Hence, the results provide viable grounds for the analyses of the impact of the techniques and solvents applied on the fatty acid composition of both extracts.

The fatty acid compositions of both extracts are displayed in Table 4. The results obtained demonstrate that the fatty acid composition of both extracts showed variations influenced by solvent polarity. Saturated fatty acids (SFAs) were slightly higher in the MAE extract (13.5%) compared to the *n*-hexane extract (12.3%), mainly due to the increased levels of myristic and palmitic acids. Monounsaturated fatty acids (MUFAs), dominated by oleic acid (9-18:1), were more abundant in the Soxhlet extract (23.0%), reflecting the higher solubility of neutral and non-polar lipids in n-hexane. In contrast, polyunsaturated fatty acids (PUFAs), primarily linoleic (18:2) and α-linolenic (18:3) acids, were slightly higher in the MAE (66.1%) than in the n-hexane (64.7%) extract.

This suggests that the polar ethanol/water mixture was more effective in extracting both free and bound lipid fractions, including polar lipids rich in essential fatty acids.

Overall, linoleic acid was the predominant fatty acid in both extracts, accounting for over 60% of the total fatty acid profile. The calculated high commensurable PUFA/SFA ratios of both extracts indicate favorable nutritional quality, although the higher MUFA content in the Soxhlet *n*-hexane oil may offer improved oxidative stability. These findings highlight that solvent polarity significantly influences lipid class recovery and fatty acid composition in oil extraction.

In a recent article [24] where the impact of roasting conditions of C. cherry stones on their valorization was reported, it was underlined that, although C. cherry stones fat content is over five times higher than that of the flesh, their chemical composition is barely described in the literature.

For example, Kucharska et al. (2009) [34] analyzed the composition of C. cherry seed oils from different cultivars in Poland and reported that they contained (in mol %) linoleic acid (70.7–75.0%), oleic acid (15.0–16.7%), stearic acid (3.5–6.2%), palmitic acid (3.5–4.6%), and linolenic acid (1.3–2.1%).

Jakovljevic et al. [30] applied SC-CO_2_ at 300 bar and 40 °C, and reported the following fatty acid composition of the oil recovered, expressed as percentage (%) of individual fatty acids to total fatty acids: palmitic acid—8.05%; stearic acid—1.93%; oleic acid—23.69%; linoleic acid—65.73%; linolenic acid—n.d.; arachidic acid—0.13%; and erucic acid—0.48%.

Vidrih et al. [26] identified six fatty acids in the seed oils of the examined ten C. cherry genotypes. The group of SFAs included palmitic, stearic, and arachidic acids, while the dominant unsaturated acids were linoleic and oleic acids. Linolenic acid was also identified but in very small quantities. Depending on the genotype, the percentage (% total fatty acids) of linoleic, oleic, and linolenic acids were in the range of 64.8 to 72.2%, 17.7–22.9%, and 1.5–1.6%, respectively.

Our results are in a good agreement with those reported in the articles above, namely the same trends in the fatty acid compositions are observed—the highest amounts observed are for linoleic and oleic acids. Still, there are some deviations in the relative percent of some of the acids. This is understandable taking into consideration the impact of many factors like genetic differences among plant varieties, environmental and climatic conditions (such as temperature, rainfall, or soil composition), harvesting or post-harvest handling practices, etc. Still, the overall fatty acid pattern identified by us and the other authors is similar.

One additional interesting comparison is that with the work of Antoniewska-Krzeska et al. [35], who examined the fatty acid composition of the different morphological parts of C. cherry, including that of seeds as well. For the latter, the authors report PUFA:SFA = 3.57.

As is known, for a balanced diet and the prevention of some socially significant diseases such as cardiovascular diseases, the PUFA:SFA ratio recommended by the British Department of Health (BDH) should be higher than 0.45. The PUFA:SFA calculated in our study for the seed extracts recovered by the MAE = 4.9 and that for Soxhlet *n*-hexane = 5.26, which are over 11 times higher than the BDH recommendation and are about 1.4 times higher than those calculated in Ref. [35].

The results displayed in Table 4 show that the composition of the extracts recovered by the two techniques is similar. Still, the MUFA value and the SFA-PUFA ratio of the Soxhlet n-hexane extract are higher than those for the MAE (route 1). However, it should be underlined that the MAE yield is over four times higher than that of the Soxhlet *n*-hexane extraction and was obtained for just 4.5 min, while in the latter case, it was obtained for 4 h.

#### 2.3.2. HPLC–HRMS/MS

HPLC–HRMS/MS was used to characterize the MAE extracts from Route 1 (MAE1) and Route 2 (MAE2). The analyses were performed using an Orbitrap mass analyzer operating in the negative electrospray ionization (ESI–) mode.

**Figure 4:**
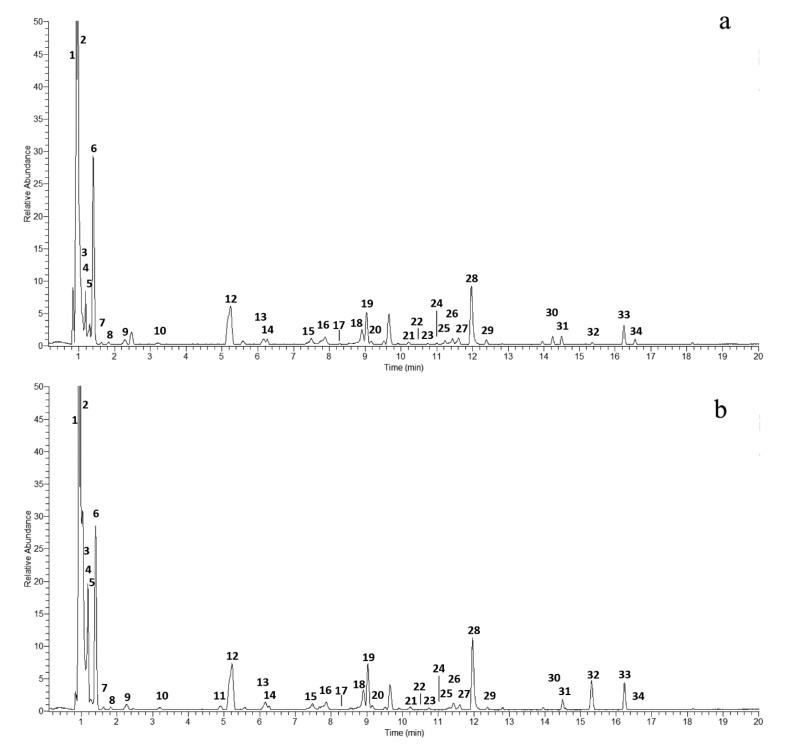
HPLC–HRMS/MS chromatogram of C. cherry stone extracts **MAE1** (**a**) and **MAE2** (**b**).

Figure 4 shows the HPLC–HRMS/MS chromatogram of C. cherry seed extracts MAE1 (a) and MAE2 (b).

A total of 34 compounds were identified based on accurate mass, isotopic patterns, and MS/MS fragmentation data, supported by the comparison with literature data and authentic standards (Table 5). Both extracts exhibited very similar qualitative profiles, differing primarily in the relative abundance of certain constituents. Hydrolysable tannins dominated the chromatographic profile but also contained notable amounts of iridoid glycosides and organic acids (Table 5).

Early-eluting peaks (0.9–1.2 min) corresponded to quinic (**1**, *m*/*z* 191.0551), malic (**2**, *m*/*z* 133.0129), and citric acids (**3**, *m*/*z* 191.0189) as [M–H]^−^ ions. These low-molecular-weight organic acids were confirmed by comparison with authentic standards.

The main constituents were hydrolysable tannins, classified as gallotannins and ellagitannins. This chemical pattern closely aligns with the findings reported in [23], who identified 37 tannins in *C. mas* stones using UPLC–Q-TOF–MS/MS. Mono-, di-, tri-, tetra-, and penta-O-galloyl-β-D-glucose derivatives were identified by characteristic neutral losses of 152 Da (galloyl residue) and fragment ions at *m*/*z* 169 (gallic acid) and 125 (decarboxylated gallate). Among the ellagitannins, gemin D, camptothin A, cornusiin A, tellimagrandin II, and their isomers were detected. These compounds exhibited typical fragment ions at *m*/*z* 301 and 275 corresponding to ellagic acid (EA) and its dehydrated forms.

Compound **17** displayed a deprotonated pseudomolecular ion at *m*/*z* 469 [M–H]^−^ and was identified as valoneic acid dilactone with fragment ions at *m*/*z* 425 [M–44 (CO_2_)-H]^−^ and 301 [EA–H]^−^. Notably, the extracts also contained free gallic (**6**) and ellagic (**28**) acids, both of which were detected with relatively high intensities.

In addition to polyphenols, three iridoid glycosides—loganic acid (**12**, [M–H]^−^, *m*/*z* 375.1297), loganin (**19**, [M+HCOO]^−^, *m*/*z* 435.1508), and cornuside (**33**, [M–H]^−^, *m*/*z* 541.1565)—were identified in both extracts. The presence of these compounds in C. cherry stones is consistent with the previous reports by Spychaj et al. [29], confirming that iridoids are retained within the stone matrix in addition to the fruit pulp. The current results demonstrate that hydroalcoholic MAE conditions enable the efficient extraction of both iridoid glycosides and polyphenolic compounds, highlighting the complementary contribution of these constituents to the overall extract composition.

Both extracts shared nearly identical compositions and contained the same series of organic acids, hydrolysable tannins, and iridoid glycosides, thus confirming a consistent metabolic profile. The only pronounced variations concerned compounds **24**, **30**, and **34**, which showed higher relative intensities in the MAE1 extract, whereas compound **32** was considerably more intense in MAE2. In addition, methyl gallate (**11**) was detected exclusively in the latter. It should be noted that methyl gallate was not identified in the Soxhlet ethanol extracts analyzed in [23], but it was reported in C. cherry unripe fruits [36]. These differences were reflected in the chromatographic profiles and further supported by comparative peak area evaluation (see Supplementary Materials, Table S1), suggesting variations in extraction efficiency or compound solubility rather than genuine compositional changes.

The presence of methyl gallate identified only in the MAE2 extract can substantiate to some extent the observed higher AA of MAE2 when compared to MAE1. Methyl gallate is a potent antioxidant that exhibits a wide spectrum of biological activities, such as anti-tumor, anti-inflammatory, antioxidant, neuroprotective, hepatoprotective, anti-HIV, etc. [37]. However, it is likely that other bioactive constituents (e.g., loganin) and/or compound **32** (unidentified) also contribute to the higher values of TPC, TFC, TSC, and DPPH, calculated for MAE2 when compared to MAE1.

The pronounced diversity and abundance of hydrolysable tannins demonstrate that C. cherry stones represent a rich and underutilized source of antioxidant polyphenols. These findings are consistent with earlier reports and reinforce the potential of stone ex-tracts for nutraceutical, pharmaceutical, and cosmetic applications, supporting sustainable approaches to *Cornus* fruit processing.

**Table 5:** Data from the HPLC-HRMS/MS analysis of C. cherry seed MAE1 and MAE2 extracts.

Peak	Rt (min)	Compound	Molecular Formula	MW (Da)	*m*/*z*	Ion Type	Δppm	Fragments	MAE1	MAE2	Ref.
1	0.93	Quinic acid *	C_7_H_12_O_6_	192.0624	191.0551	[M−H]^−^	−5.21	191, 173	+	+	
2	0.97	*L*-Malic acid *	C_4_H_6_O_5_	134.0202	133.0129	[M−H]^−^	−10.09	133, 115, 71	+	+	
3	1.20	*L*-Citric acid *	C_6_H_8_O_7_	192.0262	191.0189	[M−H]^−^	−4.28	191, 111, 87	+	+	
4	1.20	Mono-*O*-galloyl-β-d-glucose	C_13_H_16_O_10_	332.0745	331.0673	[M−H]^−^	0.56	331, 211, **169**, 125	+	+	[23]
5	1.36	Gemin D (1)	C_27_H_22_O_18_	634.0812	633.0737	[M−H]^−^	0.61	633, **301**, 275, 169, 125	+	+	[23]
6	1.40	Gallic acid *	C_7_H_6_O_5_	170.0204	169.0131	[M−H]^−^	−6.59	169, 125	+	+	
7	1.63	Gemin D (2)	C_27_H_22_O_18_	634.0812	633.0737	[M−H]^−^	0.92	633, **301**, 275, 169, 125	+	+	[23]
8	1.83	Di-*O*-galloyl-β-d-glucose	C_20_H_20_O_14_	484.0854	483.0781	[M−H]^−^	1.83	483, 331, **169**, 125	+	+	[23]
9	2.28	Camptothin A (1)	C_61_H_46_O_40_	1418.1581	708.0718	[M−2H]^−2^	1.09	785, 765, 633, **301**, 275	+	+	[23] [38]
10	3.21	Camptothin A (2)	C_61_H_46_O_40_	1418.1581	708.0718	[M−2H]^−2^	1.09	765, 633, **301**, 275, 169	+	+	[23] [38]
11	4.70	Methyl gallate	C_8_H_8_O_5_	184.0362	183.0289	[M−H]^−^	−5.33	**183**, 168, 124	-	+	[39]
12	5.23	Loganic acid	C_16_H_24_O_10_	376.1370	375.1297	[M−H]^−^	0.00	375, **213**, 169	+	+	[29]
13	6.17	Cornusiin A (1)	C_68_H_50_O_44_	1570.1695	784.0775	[M−2H]^−2^	1.29	765, **301**, 275, 169	+	+	[23]
14	6.26	Tri-O-galloyl-β-D-glucose	C_27_H_24_O_18_	636.0967	635.0894	[M−H]^−^	0.68	635, 465, 313, **169**, 125	+	+	[23]
15	7.49	Cornusiin B (1)	C_48_H_30_O_30_	1086.0827	542.0341	[M−2H]^−2^	0.50	1029, 765, **301**, 229	+	+	[23]
16	7.87	Cornusiin B (2)	C_48_H_30_O_30_	1086.0829	542.0342	[M−2H]^−2^	0.67	765, **301**, 271	+	+	[23]
17	8.27	Valoneic acid dilactone	C_21_H_10_O_13_	470.0121	469.0048	[M−H]^−^	−0.06	425, 301, **300**	+	+	[29]
18	8.90	Cornusiin A (2)	C_68_H_50_O_44_	1570.1694	784.0774	[M−2H]^−2^	1.18	935, 785, 765, **301**, 275, 169	+	+	[23]
19	9.03	Loganin	C_18_H_25_O_12_	436.1581	435.1508	[M+HCOO]^−^	0.04	**227**, 127, 101	+	+	[25]
20	9.16	Cornusiin A (3)	C_68_H_50_O_44_	1570.1698	784.0776	[M−2H]^−2^	1.49	785, 765, **301**, 275, 249, 169	+	+	[23]
21	10.21	Cornusiin A (4)	C_68_H_50_O_44_	1570.1692	784.0773	[M−2H]^−2^	1.10	935, 785, 765, 450, **301**, 275, 169	+	+	[23]
22	10.50	Cornusiin D or Camptothin B (1)	C_75_H_54_O_48_	1722.1797	860.0826	[M−2H]^−2^	0.74	937, 785, 765, **301**, 275, 169	+	+	[23]
23	10.74	Cornusiin D or Camptothin B (2)	C_75_H_54_O_48_	1722.1806	860.0830	[M−2H]^−2^	1.22	937, 785, 765, **301**, 275, 169	+	+	[23]
24	10.99	Unidentified A	C_41_H_32_O_28_	972.1084	485.0469	[M−2H]^−2^	0.36	824, 662, 265, **169**, 125	+	traces	
25	11.23	Tellimagrandin II (1)	C_41_H_30_O_26_	938.1028	937.0964	[M−2H]^−2^	1.60	838, 782, 392, 301, **169**, 125	+	+	[23]
26	11.43	Cornusiin A (5)	C_68_H_50_O_44_	1570.1697	784.0776	[M−2H]^−2^	1.40	935, 785, 765, **301**, 275, 249, 169	+	+	[23]
27	11.60	Tellimagrandin II (2)	C_41_H_30_O_26_	938.1027	937.0936	[M−2H]^−2^	−1.38	799, 392, 301, **169**, 125	+	+	[23]
28	11.96	Ellagic acid *	C_14_H_6_O_8_	302.0061	300.9988	[M−H]^−^	−0.68	301	+	+	
29	12.38	Tetra-*O*-galloyl-β-d-glucose	C_34_H_28_O_22_	788.1081	393.0467	[M−2H]^−2^	1.06	617, 317, **169**, 125	+	+	[23]
30	14.24	Ellagitannin derivative	C_41_H_30_O_27_	954.0977	476.0416	[M−2H]^−2^	0.25	597, 301, 247, **169**, 125	+	traces	[40]
31	14.50	Penta-*O*-galloyl-β-d-glucose	C_41_H_32_O_26_	940.1182	939.1119	[M−H]^−^	−1.60	769, 617, 447, 295, **169**, 125	+	+	[23]
32	15.32	Unidentified B	C_40_H_30_O_26_	926.1034	462.0444	[M−2H]^−2^	0.29	169, 125	+	+	
33	16.24	Cornuside	C_24_H_30_O_14_	542.1637	541.1565	[M−H]^−^	0.34	541, **169**, 125	+	+	[29]
34	16.56	Unidentified C	C_41_H_34_O_26_	942.1344	470.0598	[M−2H]^−2^	0.92	587, 378, 247, **169**, 125	+	+	

Rt—retention time; MW—molecular weight (monoisotopic mass). * Compounds confirmed with authentic standards.

## 3. Materials and Methods

### 3.1. Reagents and Standards

Trolox (98%), gallic acid (97.5–102.5%), sulfuric acid (H_2_SO_4_, 95–98%), 2,2′-Azino-bis (3-ethylbenzothiazoline-6-sulfonic acid) (ABTS), potassium persulfate 99% (K_2_S_2_O_8_), sodium carbonate anhydrous (Na_2_CO_3_; 99.5%), 2,2-diphenyl-1-picrylhydrazyl (DPPH), *n*-hexane, Folin–Ciocalteu reagent, 2 N, (+)-catechin hydrate ≥ 98%, and vanillin (99%) were purchased from Sigma Aldrich (St. Louis, MO, USA). Aescin (98%) was delivered from Thermo Scientific (Bremen, Germany). Ethanol (99%+) and methanol (99.9%) were from Fisher Chemical (Waltham, MA, USA), and Millipore water was obtained from the laboratory system.

The chemicals used for the GC–FID analyses were enumerated in one of our earlier works [41] and will not be given here again.

All reagents and organic solvents used in the HPLC analyses were of LC-MS-grade. Acetonitrile and methanol (Chromasolv^®^) were obtained from Honeywell Riedel-de Haën (Seelze, Germany). Formic acid was sourced from Sigma-Aldrich (Buchs, Switzerland). Ultrapure water was generated using a Smart2Pure 12 UV/UF system (Thermo Electron LED GmbH, Langenselbold, Germany). Authentic standards of quinic acid (≥98%), gallic acid (≥95%), and ellagic acid (≥95%) were obtained from PhytoLab GmbH & Co. KG (Vestenbergsgreuth, Germany).

### 3.2. Raw Material

Fruits from the wild C. cherry tree were collected from the region of Velingrad, Bulgaria. The stones were manually separated from the fruit pulp, dried at 50 °C, ground to particles smaller than 1 mm, and sieved through a 1 mm sieve.

A Thermogravimetric balance, Kern MRS 120-3 (KERN & Sohn GmbH, D-72336 Balingen, Germany), was used to measure the moisture content of biomass before (4.93 ± 0.17%) and after the n-hexane Soxhlet extraction (3.69 ± 0.18%). Measurements were performed in triplicate.

### 3.3. Sample Preparation

Two different extraction techniques, namely Soxhlet extraction and MAE, were used to recover bioactive and valuable compounds from C. cherry stones.

### 3.4. Soxhlet Extraction

The Soxhlet n-hexane extraction was performed in a Soxhlet apparatus ISOLAB NS29/32 + 34/35 (Merck KGaA, Darmstadt, Germany) on 5 g of biomass, at a liquid/solid ratio of 30/1 (v/m), until the complete discoloration of the solvent, which in our case took 4 h. In our preliminary experiments carried out at liquid/solid ratios 15:1 and 30:1, it was demonstrated that the latter led to a higher yield. The liquid extracts were concentrated using a Hei-VAP Rotary Evaporator (Heidolph Instruments GmbH & Co. KG, Schwabach, Germany) and subsequently dried in a conventional oven at 50 °C.

### 3.5. Microwave-Assisted Extraction

The CEM Discover SP microwave reactor (2.45 GHz, 300 W) (CEM Corporation, Matthews, NC, USA), described in detail in [32], was used for the extraction of bioactive substances from C. cherry stones.

For MAE, 1 g of ground C. cherry stones was placed in a 35 mL pressure vessel containing varying amounts of solvent (a mixture of water and ethanol at different ethanol concentrations (*v*/*v*)). The suspension was irradiated for 4.5 min at 70 °C, using a microwave power of 90 W with stirring. The liquid extracts were filtered through a Whatman filter, concentrated using a vacuum rotary evaporator, and then dried on a vacuum line to a constant weight.

### 3.6. Total Polyphenol Content

The total polyphenol content of the C. cherry extracts was determined using the Folin–Ciocalteu reagent in a microplate reader (BioTek Synergy 2, Winooski, VT, USA). The method was previously described in detail in Ref. [42]. The results are reported as millimoles of gallic acid equivalent per 100 g of extract (mmol GAE/100 g), based on a gallic acid calibration curve obtained under the same conditions within a concentration range of 0 to 60 µg.

### 3.7. Total Flavonoid Content

The total flavonoid content of the C. cherry stones extracts was measured using the aluminum chloride colorimetric assay [43,44]. The samples were prepared by dissolving the dry extract in 70% ethanol. Each sample (25 µL) was diluted with 100 µL distilled water, followed by the addition of 7.5 µL of a 5% (*w*/*v*) NaNO_2_ solution. After 5 min at room temperature, 7.5 µL of a 10% AlCl_3_ ethanol solution was added and left for another 5 min. Subsequently, 100 µL of a 4% (*w*/*v*) NaOH solution was added and incubated for 10 min at room temperature. The absorbance was measured at a wavelength of 415 nm in a microplate reader (BioTek Synergy 2, Winooski, VT, USA). The results were expressed as mmol of catechin equivalents per 100 g of extract (mmol CE/100 g), based on a catechin calibration curve, and measured under the same conditions within a concentration range of 0 to 200 µg/mL.

### 3.8. Total Saponin Content

The well-known vanillin–sulfuric acid method with aescin as a standard was utilized to determine TSC [45]. Initially, dry extract samples were dissolved in an appropriate volume of 70% ethanol. Each sample or standard solution of 0.25 mL was combined with 0.25 mL of a previously prepared 8% (*w*/*v*) vanillin solution in ethanol, followed by the addition of 2.5 mL of 72% sulfuric acid solution. These mixtures were then incubated in a water bath at 50 °C for 20 min. Subsequently, the reaction was stopped by cooling the mixtures in an ice bath for 10 min. A volume of 0.2 mL from each sample or standard solution was transferred to a microplate, and the absorbance was measured at a wavelength of 560 nm.

Aescin standard solutions were prepared at concentrations of 0.30–20 mg/mL, analyzed as described, and a calibration curve was generated using a microplate reader (BioTek Synergy 2, Winooski, VT, USA). The resulting linear equation was used to calculate TSC in the extracts, expressed as mmol aescin equivalents per 100 g of extract (mmol AesE/100 g).

### 3.9. Assessment of Antioxidant Activity by the DPPH Method

The antioxidant activity of the extracts was assessed using the DPPH method as detailed in our previous work [32]. In brief, the dried samples were dissolved in a 70% ethanol solution. A calibration curve was prepared using Trolox as the standard. The absorbance of the samples was measured at 517 nm in a microplate reader (BioTek Synergy 2, Winooski, VT, USA) after incubation for 40 min in the dark at room temperature. The results are expressed as mmol of Trolox equivalents (TE) per 100 g of extract (mmol TE/100 g).

### 3.10. Assessment of Antioxidant Activity by the ABTS Method

A 7.5 mM ABTS solution was prepared with 25% potassium persulfate (10 mM) using demineralized water and stored in the dark for 16 h. The solution was diluted to an optical density of 0.70 ± 0.02. Then, 180 µL of this solution was mixed with 20 µL of extract in the wells. Absorbance was measured at 734 nm using a BioTek Synergy 2 microplate reader after 6 min of incubation at room temperature in the dark [32]. All measurements were performed in triplicate. The ABTS assay results of the extracts were expressed as mmol of Trolox equivalent (TE) per 100 g of extract (mg TE/g Ex), using a calibration curve for Trolox.

### 3.11. Characterization of the Extracts

#### 3.11.1. GC-FID

The fatty acid composition of MAE Route 1 and Soxhlet *n*-hexane extracts was determined by GC-FID. Details of the method are given elsewhere [46] and will not be repeated here.

#### 3.11.2. HPLC–HRMS/MS Analysis

High-performance liquid chromatography coupled with high-resolution mass spectrometry (HPLC–HRMS/MS) was carried out using a Q Exactive Plus^®^ hybrid quadrupole-Orbitrap^®^ instrument equipped with a Vanquish UHPLC system (Thermo Fisher Scientific, Bremen, Germany). Chromatographic separation was performed on an Accucore™ C18 analytical column (150 × 2.1 mm, 2.6 µm particle size; Thermo Fisher Scientific, Germany).

The mobile phase consisted of solvent A (water containing 0.1% formic acid, *v*/*v*) and solvent B (acetonitrile). The gradient elution program was as follows: 0–2 min, 5% B; 2–25 min, 5–30% B; 25–30 min, 30–95% B; 30–34 min, 95% B; 34–35 min, 95–5% B; and 35–40 min, 5% B. The flow rate was maintained at 0.3 mL/min with an injection volume of 3 µL. Samples were initially dissolved in methanol/water (5000 mg/L) and subsequently diluted with the same solvent mixture to a final concentration of 400 mg/L.

The heated electrospray ionization (HESI) parameters were optimized as follows: spray voltage: 2.90 kV; capillary temperature: 320 °C; sheath gas flow: 30 arbitrary units; auxiliary gas flow: 6 arbitrary units; sweep gas flow: 0 arbitrary units; and S-Lens RF level: 50 V. Nitrogen was employed both as a nebulizing and as a collision gas within the HCD cell. Full-scan MS spectra were collected in the negative ionization mode over an *m*/*z* range of 120–1200, with a resolution of 70,000, an AGC target of 1 × 10^6^, and a maximum injection time of 80 ms.

Compound identification was performed using data-dependent MS^2^ (ddMS^2^) in the Top 5 mode under the following settings: resolution: 17,500, AGC target: 1 × 10^5^, maximum injection time: 50 ms, isolation window: 2.0 *m*/*z*, and stepped normalized collision energies: 20, 40, and 70. Data acquisition and analysis were conducted using the Xcalibur software (version 4.2 SP1) and FreeStyle (version 1.5), both from Thermo Fisher Scientific.

## 4. Conclusions

The MAE, a green, efficient, and sustainable technique, which is at the forefront of extraction methods, was incorporated in a two-route hybrid strategy applied for C. cherry stone biomass transformation into bioactives and other high-value compounds. In Route 1, CCD was used for the process optimization in terms of extraction yield, TFC, and TSC. In Route 2, Soxhlet n-hexane and MAE were applied consecutively.

MAE1 and Soxhlet *n*-hexane extracts yielded similar fatty acid profiles, dominated by linoleic and oleic acids. The MAE favored PUFA-rich polar lipids, while Soxhlet enriched MUFA. Both extracts exhibited high PUFA/SFA ratios, indicating excellent nutritional quality. The MAE achieved comparable recovery in just 4.5 min, compared with 4 h for Soxhlet, demonstrating superior extraction efficiency.

The HPLC–HRMS/MS analysis showed that both MAE1, and MAE2, recovered in stage 2 of Route 2, are rich in hydrolysable tannins, iridoid glycosides, and organic acids. Still, the TPC, TFC, and TSC values calculated for MAE2 are higher than those of MAE1, with the ABTS value of the former being about 1.7 higher. This enhanced antioxidant activity is most likely linked to the higher overall phenolic content of MAE2, particularly the presence of methyl gallate (which was not detected in MAE1) as well as subtle differences in the relative abundance of other bioactive constituents.

These findings demonstrate that the CCD-optimized hybrid strategy effectively maximizes the recovery of bioactive compounds, highlighting the superior potential of Route 2 for obtaining antioxidant-rich extracts from C. cherry stones. Furthermore, they mark the future directions to boosting the C. cherry seed biomass transformation into high-value compounds with applications in food, nutraceutical, pharmaceutical, and cosmetic industries.

## Figures and Tables

**Table 1:** Range of examined parameters.

Factor	Name	Units	Minimum	Maximum	Coded Low	Coded High
A	Ratio (solv/sol)	mL/g	7.90	22.10	−1 ↔ 10.00	+1 ↔ 20.00
B	Ratio (Eth/wat)	%	5.50	104.50	−1 ↔ 20.00	+1 ↔ 90.00

**Table 2:** CCD test results and the response functions evaluated in each experiment.

A-Ratio Solv/Sol	B-Ratio Eth/Wat	Yield	TPC	DPPH	ABTS	TFC	TSC
mL/g	%	%	(mmol GAE/100 g)	(mmol TE/100 g)	(mmol TE/100 g)	(mmol CE/100 g)	(mmol AesE/100 g)
10	20	15.84	41.48 ± 1.36	205.76 ± 2.58	178.59 ± 4.89	45.46 ± 1.36	9.97 ± 0.79
20	20	17.90	47.01 ± 0.73	213.37 ± 2.17	205.52 ± 5.00	55.32 ± 4.77	11.68 ± 1.00
10	90	17.04	49.44 ± 1.55	215.06 ± 3.46	200.70 ± 2.58	63.75 ± 6.76	12.33 ± 0.46
20	90	17.17	42.70 ± 0.45	214.93 ± 2.08	178.403 ± 3.48	44.86 ± 4.08	10.91 ± 1.07
7.9	55	14.39	45.03 ± 0.29	204.86 ± 4.24	192.58 ± 3.62	62.06 ± 6.39	10.18 ± 0.16
22.1	55	18.78	48.48 ± 2.20	251.13 ± 3.67	231.85 ± 10.28	54.27 ± 1.53	11.07 ± 0.40
15	5.5	18.97	50.46 ± 3.11	207.99 ± 1.37	201.25 ± 4.19	48.09 ± 2.60	11.44 ± 0.13
15	100	15.60	45.96 ± 0.53	205.98 ± 2.41	189.062 ± 3.52	46.56 ± 2.85	10.49 ± 0.23
15	55	18.13	40.27 ± 1.64	205.56 ± 2.85	184.59 ± 6.96	42.62 ± 1.79	11.92 ± 0.80
15	55	17.76	46.99 ± 2.85	205.25 ± 2.67	199.07 ± 3.38	49.87 ± 6.12	12.31 ± 0.52
15	55	16.78	47.39 ± 1.86	231.04 ± 2.77	208.26 ± 4.41	55.12 ± 2.49	12.94 ± 0.48
15	55	17.70	50.56 ± 1.27	217.75 ± 2.54	214.53 ± 2.54	53.55 ± 2.52	12.73 ± 0.78
15	55	15.41	51.69 ± 2.59	219.63 ± 2.83	214.53 ± 5.20	55.92 ± 8.35	13.37 ± 0.68

**Table 3:** Results of the MAE performed on the dried residual matrix, after the Soxhlet *n*-hexane extraction (Route 2, stage 2).

A-Ratio Solv/Sol	B-Ratio Eth/Wat	Yield	TPC	DPPH	ABTS	TFC	TSC
mL/g	%	%	(mmol GAE/100 g)	(mmol TE/100 g)	(mmol TE/100 g)	(mmol CE/100 g)	(mmol AesE/100 g)
15	5	11.82 ± 0.77	55.58 ± 1.53	230.59 ± 5.12	316.50 ± 21.14	56.40 ± 3.82	15.16 ± 1.17
15	55	17.90 ± 1.05	60.31 ± 1.89	245.98 ± 1.92	330.38 ± 11.93	62.88 ± 3.89	10.30 ± 0.26
15	100	11.24 ± 0.60	55.20 ± 1.77	242.42 ± 5.70	320.98 ± 20.96	59.10 ± 5.21	12.74 ± 0.40

**Table 4:** Fatty acid composition from the FAME GC-FID analysis of C. cherry stones’ MAE extract in the central point and Soxhlet extracts, expressed as the relative percent of total fatty acids identified.

Fatty Acid, Rel.%	MAE	Soxhlet *n*-Hexane
Myristic, C14:0	0.20 ± 0.05	0.10 ± 0.05
Pentadecanoic, C15:0	0.10 ± 0.05	n.d.
Palmitic, C16:0	9.0 ± 0.1	8.0 ± 0.1
Margaric acid, C17:0	0.20 ± 0.05	0.10 ± 0.05
Stearic acid, C18:0	2.20 ± 0.05	2.3 ± 0.1
Oleic acid, C9-18:1	19.5 ± 0.1	22.1 ± 0.1
Trans vaccenic acid, C11-18:1	0.40 ± 0.05	0.40 ± 0.05
Linoleic acid, C18:2	64.7 ± 0.4	63.5 ± 0.3
α-Linolenic acid, C18:3	1.4 ± 0.0	1.2 ± 0.1
Arachidic acid, C20:0	0.30 ± 0.05	0.40 ± 0.05
Eicosenoic acid, C20:1	0.4 0 ± 0.05	0.50 ± 0.05
Behenic acid, C22:0	1.0 ± 0.1	0.9 ± 0.1
Lignoceric acid, C24:0	0.60 ± 0.05	0.5 ± 0.1
SFA	13.5	12.3
MUFA	20.3	23.0
PUFA	66.1	64.7
PUFA:SFA	4.90	5.26

## Data Availability

Data are contained within the article.

## Supplementary Materials

The following supporting information can be downloaded at: https://www.mdpi.com/article/10.3390/molecules31030525/s1, Table S1. Peak areas of compounds in C. cherry MAE extracts obtained by HPLC-HRMS/MS.

## Abbreviations

The following abbreviations are used in this manuscript:

MAE	microwave-assisted extraction
CCD	central composite design
GC-FID	gas chromatography–flame ionization detection
HPLC–HRMS/MS	high-performance liquid chromatography–high-resolution mass spectrometry
FAME	fatty acid methyl ester
TPC	total polyphenol content
GAE	gallic acid equivalent
TFC	total flavonoid content
AA	antioxidant activity
DPPH	2,2-diphenyl-1-picrylhydrazyl
ABTS	2,2′-azino-bis(3-ethylbenzothiazoline-6-sulfonic acid
TE	trolox equivalent
TSC	total saponin content
AesE	aescin equivalent
CI	confidence interval
QE	quercetin equivalent
FRAP	ferric reducing ability of plasma
SFA	saturated fatty acid
MUFA	monounsaturated fatty acid
PUFA	polyunsaturated fatty acid
SC-CO_2_	supercritical CO_2_
UPLC-Q-TOF-MS/MS	ultra-performance liquid chromatography–quadrupole time-of-flight mass spectrometry
HESI	heated electrospray ionization

## Appendix A

**Table A1:** ANOVA results of the selected CCD models. Estimated regression model of the relationship between a response variable and the independent variables.

	Yield (%)				TFC (mmol CE/100g)				TSC (mmol AesE/100g)			
Source	SS ^a^	MS ^b^	F-Value	*p*-Value	SS ^a^	MS ^b^	F-Value	*p*-Value	SS ^a^	MS ^b^	F-Value	*p*-Value
Model	11.14	5.57	4.73	0.0358	382-38	76.48	4.07	0.0473	11.21	2.24	5.60	0.0215
A-Ratio (Solv/sol)	8.83	8.83	7.50	0.0209	50.25	4.01	2.67	0.1460	0.3006	0.3006	0.7508	0.4149
B-Ratio (Eth/water)	2.31	2.31	1.96	0.1918	4.01	4.01	0.2135	0.6580	0.0076	0.0076	0.0190	08944
AB					206.64	206.64	11.00	0.0128	2.45	2.45	6.12	0.0426
A^2^					74.66	74.66	3.97	0.0865	5.74	5.74	14.35	0.0068
B^2^					31.78	31.78	1.69	0.2346	3.80	3.80	9.50	0.0178
Residual	11.77	1.18			131.55	18.79			2.80	0.4003		
Lack of Fit	6.97	1.16	1.79	0.9684	12.23	4.41	0.1491	0.9251	1.55	0.5150	1.64	0.3151
Pure Error	4.80	1.20			118.32	29.58			1.26	0.3143		
Cor Total	22.91				513.94				14.02			

^a^ Sums of squares. ^b^ Mean square.
