# Supplementary material for: Boosting the Sustainable Transformation of Cornus mas L. Stones Using a Hybrid Strategy Involving Microwave-Assisted Extraction
Source: Molecules. 2026 Feb 2;31(3):525. doi: 10.3390/molecules31030525 (PMC12899463; doi:10.3390/molecules31030525)
Supplement: Supplementary file 1 [file molecules-31-00525-s001.zip › molecules-4029560-supplementary.pdf]

Supplementary Materials

Table S1. Peak areas of compounds in C. cherry MAE extracts obtained by HPLC-HRMS/MS

Peak	Rt (min)	Compound	Area in MAE1	Area in MAE2
1	0.93	Quinic acid	1E+09	1E+09
2	0.97	*L*-Malic acid	1E+09	6E+08
3	1.20	*L*-Citric acid	2E+07	1E+07
4	1.20	Mono-*O*-galloyl-β-*D*-glucose	5E+07	7E+07
5	1.36	Gemin D (1)	2E+07	3E+07
6	1.40	Gallic acid	6E+08	6E+08
7	1.63	Gemin D (2)	2E+07	2E+07
8	1.83	Di-*O*-galloyl-β-*D*-glucose	2E+07	2E+07
9	2.28	Camptothin A (1)	5E+07	6E+07
10	3.21	Camptothin A (2)	5E+07	5E+07
11	4.90	Methyl gallate	-	4E+07
12	5.23	Loganic acid	3E+08	3E+08
13	6.17	Cornusiin A (1)	8E+07	1E+08
14	6.26	Tri-O-galloyl-β-D-glucose	5E+07	4E+07
15	7.49	Cornusiin B (1)	7E+07	7E+07
16	7.87	Cornusiin B (2)	1E+08	1E+08
17	8.27	Valoneic acid dilactone	2E+07	9E+06
18	8.90	Cornusiin A (2)	1E+08	2E+08
19	9.03	Loganin	1E+08	2E+08
20	9.16	Cornusiin A (3)	3E+07	5E+07
21	10.21	Cornusiin A (4)	3E+07	4E+07
22	10.50	Cornusiin D or Camptothin B (1)	1E+07	2E+07
23	10.74	Cornusiin D or Camptothin B (2)	3E+07	3E+07
24	10.99	Unidentified A	2E+07	-
25	11.23	Tellimagrandin II (1)	3E+07	1E+07
26	11.43	Cornusiin A (5)	7E+07	6E+07
27	11.60	Tellimagrandin II (2)	5E+07	5E+07
28	11.96	Ellagic acid	3E+08	3E+08
29	12.38	Tetra-*O*-galloyl-β-*D*-glucose	3E+07	2E+07
30	14.24	Ellagitannin	5E+07	-
31	14.50	Penta-*O*-galloyl-β-*D*-glucose	4E+07	5E+07
32	15.32	Unidentified B	9E+06	1E+08
33	16.24	Cornuside	7E+07	1E+08
34	16.56	Unidentified C	3E+07	7E+06
